# Relaxin inhibits patellar tendon healing in rats: a histological and biochemical evaluation

**DOI:** 10.1186/s12891-019-2729-3

**Published:** 2019-07-27

**Authors:** Tianpeng Xu, Jiaxiang Bai, Menglei Xu, Binqing Yu, Jiayi Lin, Xiaobin Guo, Yu Liu, Di Zhang, Kai Yan, Dan Hu, Yuefeng Hao, Dechun Geng

**Affiliations:** 1 Orthopedics and Sports Medicine Center, The Affiliated Suzhou Hospital of Nanjing Medical University, 242, Guangji Road, Suzhou, 215006 People’s Republic of China; 2 Department of Orthopedics, The First Affiliated Hospital of Soochow University, 188, shi zi Road, Suzhou, 215006 People’s Republic of China

**Keywords:** Relaxin, Female, Tendon healing, Extracellular matrix (ECM), Relaxin family peptide receptor 1(RXFP1)

## Abstract

**Background:**

Female patients are more likely to have tendon injuries than males, especially those who has a higher concentration of relaxin. Previous studies have demonstrated that relaxin attenuates extracellular matrix (ECM) formation. However, the mechanism of relaxin on tendon repair remains unclear. We hypothesize that relaxin inhibits tendon healing by disrupting collagen synthesis.

**Methods:**

A patellar tendon window defect model was established using Sprague-Dawley rats. The center of the patellar tendon was removed from the patella distal apex and inserted to the tibia tuberosity in width of 1 mm. Then, the rats were injected with saline (0.2 μg/kg/day) or relaxin (0.2 μg/kg/day) for two and four weeks, which was followed by biomechanical analysis and histological and histochemical examination.

**Results:**

Mechanical results indicated that relaxin induces a significant decrease in tear resistance, stiffness, and Young’s modulus compared to those rats without relaxin treatment. In addition, it was shown that relaxin activates relaxin family peptide receptor 1(RXFP1), disturbs the balance between matrix metalloproteinases (MMPs) and tissue inhibitors of metalloproteases (TIMPs), and reduces the deposition of collagen in injury areas.

**Conclusions:**

Relaxin impairs tendon healing in rats. Also, relaxin might lead to tendon injury more commonly for females than males.

**Electronic supplementary material:**

The online version of this article (10.1186/s12891-019-2729-3) contains supplementary material, which is available to authorized users.

## Background

Tendons, which are dense fibrous connective tissues composed of tenocytes and abundant ECM, connect muscles to bones and facilitate in the transmission of muscle-generated forces to the bones, resulting in joint motion [1]. With the increasing popularity of sports, more people now engage in physical exercise. However, due to inappropriate actions, accidents, and population aging, the morbidity of activity-related injuries such as tendon injuries has rapidly increased. There are more than 30 million tendon injuries around the world each year, and the actual number is even higher because many injuries are not reported [2]. Unfortunately, tendons have poor self-repair capability due to their nature of minimal blood supply, poor oxygen consumption, and low metabolic capacity [3]. Rui et al. showed that tendon healing is a failed healing or nonhealing process. Histologically, tendinopathic tissues exhibit a failed healing condition that is characterized by tissue metaplasia and include chondrocyte phenotypes, fatty infiltration, and bony deposits in some tendinopathy patients and animal models [4]. One of the important factors of tendon healing was ECM. Changes in the physiological conditions of ECM after tendon injury may trigger protease activities and impair the balance between MMPs and TIMPs. This imbalance further induces collagen degeneration, which is critical to tendon tissue integrity [5].

Research investigations on sex hormones have recently increased. There is a quiet difference between women and men for the risk of tendon and ligament injuries. Sex hormonal differences may one of the major reasons [6]. For example, estrogen had a negative effect on tendon healing and metabolism and reduced tendons composition like collagen I, aggrecan and elastin. Collagen I, aggrecan and elastin [6, 7]. Meanwhile, Relaxin, a polypeptide hormone that consists of relaxin 1–3 and insulin-like peptides (INSL3, 4, 5, and 6), can also affect the tendons or ligaments. The corpus luteum and the placenta mainly synthesized it. Relaxin affects the extracellular matrix components of tissues. The two main relaxin receptor isoforms include RXFP1 and RXFP2. While H1 and H2 (human relaxin) activate RXFP1 and RXFP2, rat relaxin 1 weakly binds to RXFP2 [8].

When mice are pregnant, relaxin transforms the pubic articular cartilage to a pliable interligament in adapt to the enlarged uterus. Meanwhile, relaxin itself or combined with estrogen reduce public collagen content, which is antagonized by progesterone, in non-pregnant rats [9].

It has been shown that relaxin is antifibrotic as it downregulate fibroblast activity, increase collagenase synthesis, and inhibits collagen-1 lattice contraction in rat kidneys, which is stimulated by transforming growth factor-β [10]. According to Wang et al. [11], they find that relaxin receptors are expressed in mouse knee fibrocartilage, which suggest the knee is also a potential target of relaxin. Relaxin receptors have been identified both in females and males’ anterior cruciate ligament (ACL), meanwhile there is a high expression in females. Dehghan et al. [9] reported that RXFP1 and RXFP2 are expressed in rat patellar tendon, which are upregulated by progesterone and high doses of estrogen.

Dragoo et al. [12] showed that female athletes occurred with ACL tears have higher concentration of relaxin. Those with serum relaxin concentrations > 6 pg/mL have a four-fold higher risk for tears. Athletes often develop chronic tendinopathy due to chronic tendon injuries or overuse of their tendons coupled with insufficient rest after injuries. Therefore, the probability of ACL or other tendon tears is significantly higher than in non-athletes. During sports activities, female athletes tear their ACL two to eight times more frequently than males and this may be attributable to relaxin, which influences the composition of ECM and contributes to tendon degeneration and increased risk of tendon injury in female athletes [13, 14].

However, the effect of relaxin on tendon and ligament healing remains unclear. We hypothesized that relaxin inhibits tendon healing, and carried out this study to test it in a rat patellar tendon window defect model.

## Methods

All animal procedures were approved by the Ethics Committee of the First Affiliated Hospital of Soochow University (Suzhou, Jiangsu, China).

### Animals

A total of 36 female Sprague-Dawley rats, 6–8 weeks old and weighing from 250 to 300 g, were used in this study. The rats were purchased from the Experimental Animal Center of Soochow University. Also, they were kept in a ventilated environment with a 12 h:12 h light-dark cycle at a constant temperature of 21 °C.

### Rat patellar tendon repair model

**Fig. 1:**
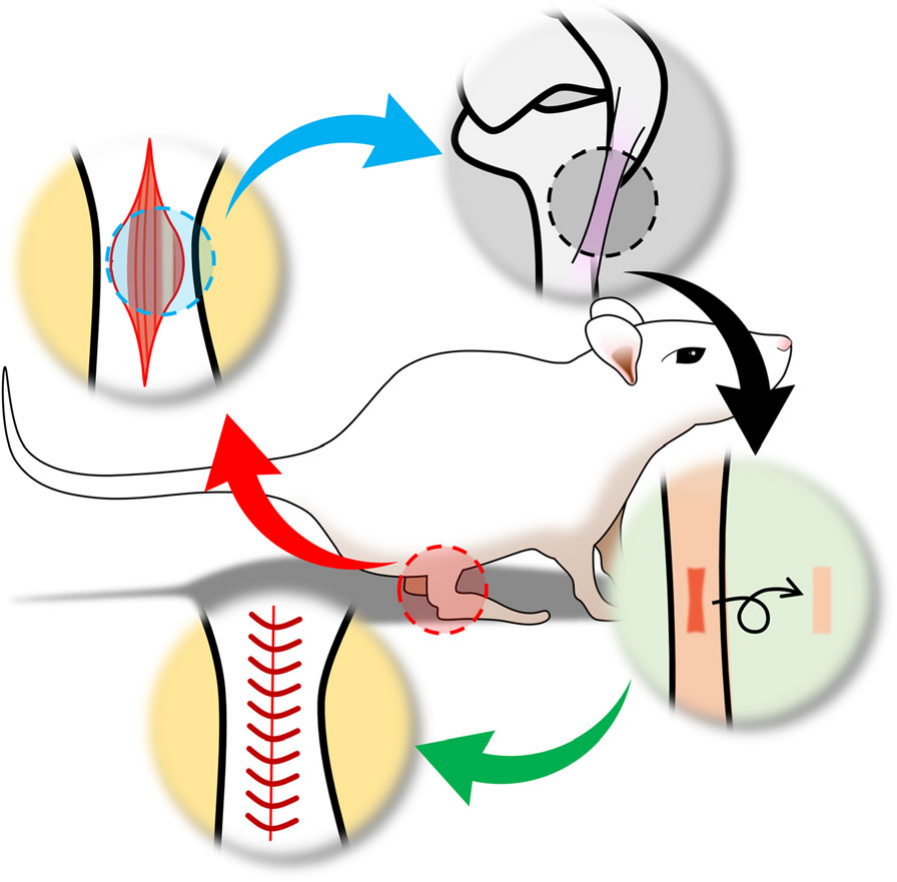
A 1 × 4 mm window defect was created in patellar tendon of rat

To induce tendon defects, two stacked sharp blades, described in a previous study, was used to remove the center of the patellar tendon (1 mm in width) from the patella distal apex and insert to the tibia tuberosity without causing any damage to the fibrocartilage zone [15] (Fig. 1). The surgery was performed under sterile conditions. After surgery, nylon 5–0 suture were used to close the skin incision. The rats were placed on heating pads in the recovery cages until they recovered from anesthesia. For the first 24 h of recovery, the animals were housed in individual cages and were provided with softened rat chow. The operated rats were divided into two groups: the vehicle group and the relaxin group, with 12 rats in each group. The 12 rats that underwent sham surgery were used as the control group. The vehicle group received subcutaneous injection near the knees of saline (0.2 μg/kg/day) for two and four weeks. The relaxin group also received subcutaneous injection of relaxin (0.2 μg/kg/day) (Protech, Nevada, USA) for two and four weeks. The injection needle was a kind of micro syringe (Gaoge, Shanghai, China). Diameter of needle is 0.5 mm. When we did the injections, two operators performed at the same time, one operator was in fixing the neck and limbs of the rats, and the other operator was responsible for the injection. At the time of injection, the needle tip of the syringe was injecteded with an angle of 30°-50° and avoided damage to surrounding skin and subcutaneous tissue. The dose of relaxin used in this study was based on previous investigations [16]. The rats were sacrificed by intraperitoneal injection an over dose of sodium pentobarbital (150 mg/kg) after 2 and four weeks.

### Biomechanical analysis

The right patellar tendons of rats, sacrificed 2 and 4 weeks post-operatively, were kept at room temperature in PBS-soaked gauze pads until testing within 1 h after collection. To carry out tensile testing of the tendons, the patella and the proximal tibia were fixed at each clamp (5 mm in between two clamps) of a testing machine (Instron, MA, USA). The samples were tested at a speed of 1 mm/s until failure.

### Histological and histochemical examination

For histological analysis, the left patellar tendon was fixed in 4% paraformaldehyde for one day. After embedding in paraffin, the samples were sectioned longitudinally at a thickness of 6 μm, then deparaffinized in xylene, washed, and hydrated with washing ethanol. The sections were processed for histological examination using hematoxylin-eosin (H&E) and Masson trichrome staining. To carry out the regenerating tissue quality evaluation, we adopted a histological scoring system [12]. The histological analysis was examined independently by two blinded observers. The scores of seven defined tissue regeneration criteria, which were described by Stoll (Additional file 1: Table S1), were then recorded [17]. A score of 12 was given to an intact Achilles tendon without any surgical operation.

The sections were de-waxed, hydrated and inactivated. Then the tissue were blocked with 5% normal goat serum after antigens repairing and incubated at 4 °C overnight using Col I (1:500), Col III (1:500) (Abcam, Shanghai, China), RXFP1 (1:200) (Biorbyt, California, USA), MMP1 (1:500), MMP9 (1:500) (Abcam, Shanghai, China) and MMP13 (1:500) (Biorbyt) monoclonal antibodies. Finally, they were incubated with HRP-conjugated secondary antibody (1:2,000) for 1 h. Sections were colored by incubating with the chromogen 3.3′-diaminobenzidine (DAB) tetrahydrochloride and counterstained with hematoxylin.

One-way ANOVA and post-hoc Bonferroni tests with SPSS 20.0 were adopted to carry out statistical analyses of histological score and biomechanical testing. Statistical significance was assigned at *p* values less than 0.05.

## Result

### Relaxin disrupts tendon healing

There was no loss of specimens during the tests. The results of mechanical testing showed that tissue repair after tendon removal at each postoperative time point in the vehicle and relaxin groups was worse than in the control group (Table 1). At two weeks after surgery, maximum load, stiffness, and Young’s modulus in both the vehicle and relaxin group were lower than in the control group (*p* < 0.05). This observation suggests that surgical operations change the mechanical properties of tendons. The results demonstrate that the vehicle and relaxin groups have similar mechanical properties during the earlier period of repair. However, the data obtained four weeks postoperatively indicated that the control group exhibited significantly higher maximum load and Young’s modulus (*p* < 0.05) than the relaxin group. These findings suggest that relaxin disrupts tendon healing, whereas the control group exhibited regeneration. No statistical difference in tendon length and cross-sectional areas between the three groups after two and four weeks was observed.

**Table 1:** Results of mechanical testing

Groups		Length (mm)	Area (mm^2^)	Maximum load (N)	Stiffness (N/mm)	Young’s modulus (MPa)
Two-week	Control	6.02 ± 0.65	4.17 ± 0.80	74.83 ± 5.87	39.93 ± 5.99	18.95 ± 1.73
	Vehicle	6.09 ± 0.66	4.77 ± 0.39	41.91 ± 11.19^a^	20.77 ± 3.40^a^	9.20 ± 2.63^a^
	Relaxin	5.09 ± 0.55	4.06 ± 0.83	39.48 ± 5.93^a^	14.71 ± 3.17^a^	5.42 ± 2.70^a^
Four-week	Control	5.95 ± 0.94	3.88 ± 0.70	76.54 ± 6.41	40.12 ± 5.82	17.66 ± 2.25
	Vehicle	6.12 ± 0.35	4.91 ± 0.78	65.90 ± 7.69	28.26 ± 7.81^a^	15.91 ± 3.77
	Relaxin	5.71 ± 0.94	4.39 ± 0.56	50.57 ± 9.91^a,b^	21.05 ± 5.96^a^	9.39 ± 3.37^a,b^

^a^ *p* < 0.05 significantly different from the control group

^b^ *p* < 0.05 significantly different from the vehicle group

### Relaxin reduces the deposition of collagen in injury areas

**Fig. 2:**
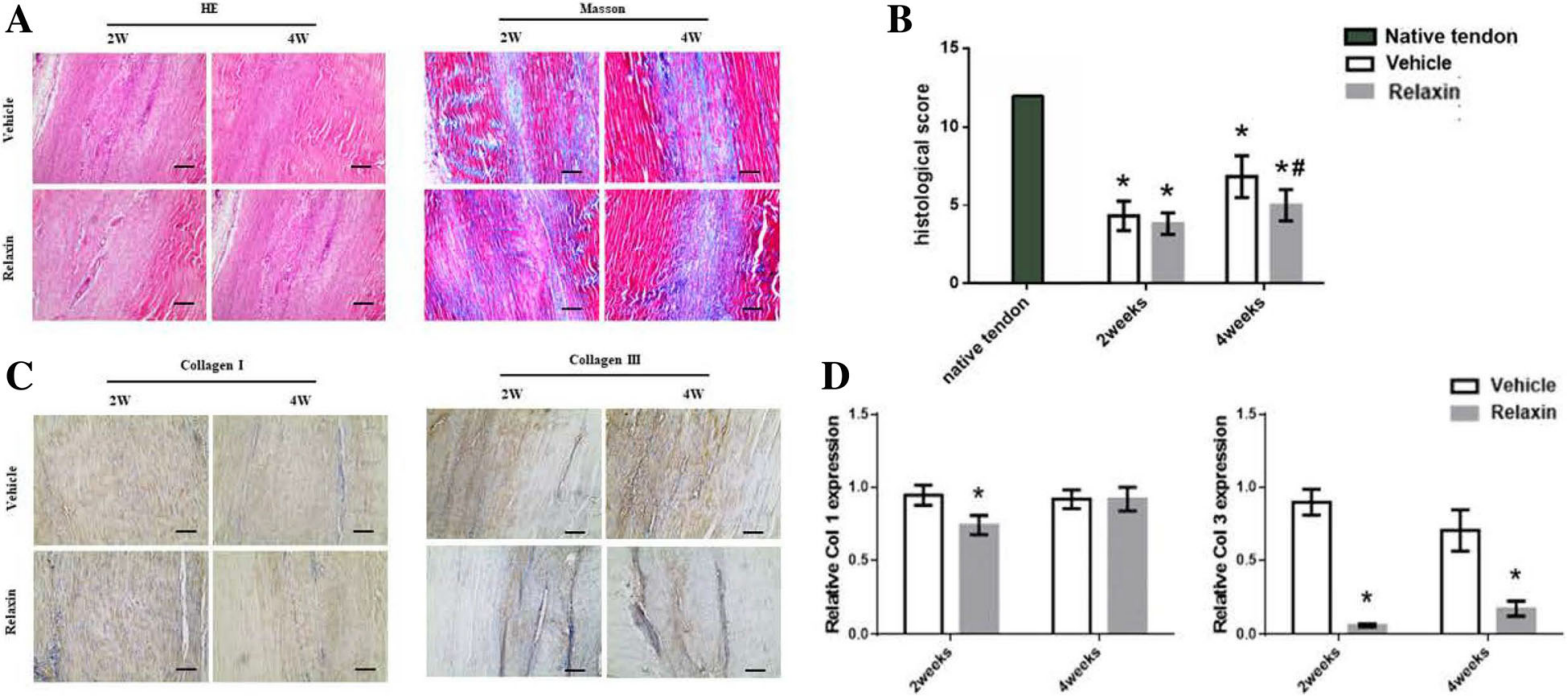
Histopathological and immunohistochemical findings of repaired tendons in the vehicle and relaxin groups. **a**: H&E and Masson staining. **b**: Histological scores at the 2nd and 4th postoperative weeks. **c**: Immunohistochemical staining of collagen I and collagen III in the tendon wound areas in the 2nd and 4th postoperative weeks. *p < 0.05 vs. native tendon, #*p* < 0.05 compared with vehicle group. (Scale bar = 200 μm). **d**: Relative expression levels collagen I and collagen III in relaxin group compared with vehicle group. *p < 0.05 compared with vehicle group

H&E staining revealed delayed healing in the relaxin group (Fig. 2a). Early collagen formation was seen two and four weeks after wounding in the vehicle and the relaxin groups, respectively. Two weeks after wounding, both vehicle and relaxin groups showed high cell density in the wound areas. However, four weeks after wounding, the vehicle group exhibited a more distinct cell arrangement than the vehicle group. Masson staining was utilized to evaluate collagen maturation levels. At week 2, there were more collagen found in vehicle group than in relaxin group. At week 4, there was further deposition observed. (Fig. 2a). At each time point, intact tendons showed significant differences in histological scores compared with both study groups (*p* < 0.05). However, the relaxin group presented lower histological scores (*p* < 0.05) than the vehicle group, indicating that the relaxin group exhibited poor tendon structural recovery (Fig. 2b). These findings indicate that relaxin impairs collagen deposition in the wound areas.

At week 2 and 4 after surgery, immunohistochemistry staining of Col I and Col III were applied in the new tendon tissues. The expressions of Col I and Col III in the neo-tendon tissues were stronger in the vehicle group and more uniform at both weeks 2 and 4. Compared to normal tendons, the expressions of Col I and III were more intense, suggesting that more ECM was deposited into the wound areas during tendon healing (Fig. 2c). The relative expression of Col I and Col III is shown in Fig. 2d, demonstrating a much higher abundance of Col III of vehicle group relative to relaxin group (*p* < 0.05).

### Relaxin changes the balance between MMPs and TIMP1 by activating RXFP1

**Fig. 3:**
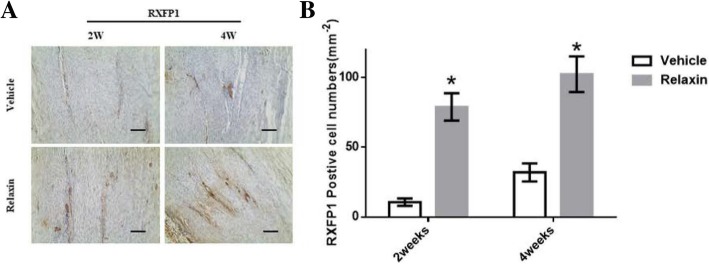
Relaxin activates RXFP1 expression. **a**: Immunohistochemical staining of RXFP1 in the tendon wound areas in the 2nd and 4th postoperative weeks. **b**: Quantification of RXPF1 positive cells expression in the tendon wound areas. *p < 0.001 compared with vehicle group. (Scale bar = 200 μm)

To investigate how relaxin alters collagen deposition in wound areas, we first evaluated the expression of RXFP1, which is the receptor for relaxin. Immunohistochemical staining showed higher RXFP1 expression in the wound areas of the relaxin group compared to the vehicle group (Fig. 3a). An increase in RXFP1 expression in the wound areas was observed in the relaxin group (*p* < 0.05) (Fig. 3b). These results reveal that relaxin increases RXFP1 expression in injured tendons.

**Fig. 4:**
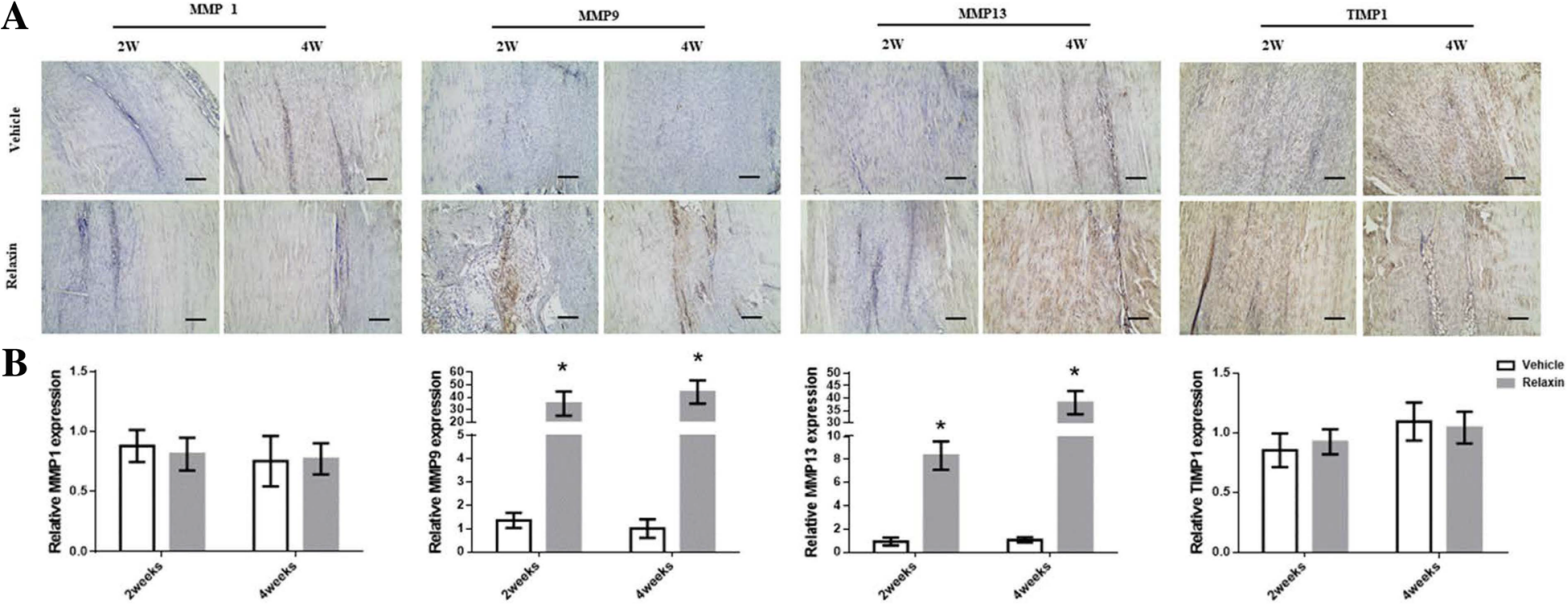
Relaxin disrupts the balance between MMPs and TIMP. **a**: Immunohistochemical staining of TIMP1, MMP1, MMP9, and MMP13 in the tendon wound areas in the 2nd and 4th postoperative weeks. (Scale bar = 200 μm). **b**: Relative expression of MMP1, MMP9, MMP13 and TIMP1 in tendon wound areas. MMP9 and MMP13 shows a significant difference in the 2nd and 4th postoperative weeks. *p < 0.001 compared with vehicle group

MMPs play an important role in collagen metabolism. We assessed the effects of relaxin on the expression of MMPs and TIMP1. Immunohistochemical staining indicated that MMP9 and MMP13 were highly expressed in the relaxin group (*p* < 0.05). However, no difference in MMP1 and TIMP1 expression between the two groups was observed. Relaxin treatment effectively increased the expression of MMP9 and MMP13. These findings indicate an alteration in the balance between MMPs and TIMP expression (Fig. 4), thereby resulting in worse healing in the relaxin group compared to the vehicle group.

## Discussion

Relaxin, a peptide hormone belonging to the insulin superfamily, is involved in the promotion of ECM remodeling. The present study has shown that rats injected with relaxin exhibited inferior healing of injured tendons.

We evaluated the effects of relaxin on the mechanical properties of tendon healing. No significant differences in length and cross-sectional areas were observed among the vehicle, relaxin, and control groups. This finding may be due to that the rats do not produce a large amount of scar tissues in the patellar tendon during injury. However, in the rat Achilles tendon injury model, alterations in cross-sectional area and length were observed that operation had caused severe scar tissue and increased cross-sectional areas after healing of achilles tendon [18]. The present study confirmed that both vehicle and relaxin groups exhibit lower maximum load, stiffness, and Young’s modulus compared to the controls. However, the vehicle group showed improvement at four weeks after surgery, whereas the maximum load and Young’s modulus of the relaxin group remained worse than the control group. These results demonstrate that relaxin disrupts the healing process, thereby resulting in weak tendons.

The histological scores of the present rat model were indicative of the degeneration of tendon mechanical properties two or four weeks after surgical removal. H&E staining showed that relaxin inhibits tendon repair (Fig. 2b). Over time, collagen deposition occurred in the wound area, as indicated by more intense Masson staining. Col I, essential for fibrous tissues repair and regeneration, is one the main ECM protein in tendon [19]. The collagen fibers longitudinal arrangement is important to provide mechanical properties of tendons and can maintain the physiological structure [20, 21]. The rat model showed that the control group had more Col I and Col III accumulation in the wound area. All mechanical and histological data indicate that repair tissues in the vehicle group, which did not receive relaxin treatment, exhibited more extensive improvement than the relaxin group.

The MMPs family is composed of more than 20 zinc-dependent metalloproteinases that are responsible for cell adhesion, migration, proliferation, and differentiation [22]. MMP1 and MMP13 are members of this family that are strongly associated with collagen degradation and metabolism [23]. In the present study, the expression of Col I and Col III in tendon ECM was significantly higher in the control group, whereas that of RXFP1 and MMP13 was upregulated in the relaxin group. These findings reveal that relaxin stimulates the expression of MMP13 by activating RXPF1, thereby altering the structure and promoting the degradation of collagen. These results imply that RXPF1 and MMPs expression in vivo may be utilized as an indicator for the risk of ligament tears.

Previous rat studies have shown that drugs, exercise, and biomaterials improve tendon healing [24–27]. Moreover, the change of sex hormone are related to tendon diseases. Ganderton et al. found that menopausal hormone therapy (MHT) could improve the pain and function in post-menopausal women with greater trochanteric pain syndrome (GTPS) [28]. Furthermore, external factors can enhance the histological and biomechanical properties of tendons. However, the regulatory mechanism underlying the inhibition of tendon repair remains elusive. Zhang al et [29]. proposed that tendon healing is a failure process because of poor blood supply and nerve growth and the imbalance of ECM. Relaxin can activate MMPs, which in turn promote collagen degradation in the fibrocartilaginous temporomandibular joint [30]. Changes in the physiological conditions of the ECM following tendon injury can trigger protease activity and compromise the balance between MMPs and TIMP. This imbalance would further induce collagen degeneration, causing poor tendon and ligament healing.

The findings of this study also showed that in rat patellar tendons, relaxin increased the expression of RXFP1 and promoted the expression of MMP9 and 13, which in turn reduced collagen deposition in the wound area. In addition, the findings of this study also identify a novel target to improve the healing of tendons.

One of the limitations of this study is that we did not investigate the effect of relaxin on tenocytes or tendon stem cells and the animal models did not include a castration group and did not assess whether relaxin has the same biological effects on castration. Secondly, it is not clear whether downstream signaling pathways activate RXFP1. Lastly, we applied a patellar ligament injury model, which is not happened in nature. This did not fully explain the healing mechanism of human tendon ligament injury.

## Conclusion

Relaxin disrupts patellar tendon healing in a rat model. Further in vitro experiments confirming the effect of relaxin on tenocytes or tendon stem cells are warranted. Although the levels of hormones in humans and rats are not the same, women exhibit more extensive fluctuations in sex hormone levels during the menstrual cycle. In addition, studies on the role of relaxin in ligament healing should be performed.

## Additional file

Additional file 1: **Table S1.** Histological scoring system. (DOCX 17 kb)

## Data Availability

The datasets used and analyzed during the current study are available from the corresponding author on reasonable request.

## Abbreviations

ACL H&E: Hematoxylin-eosin
ACL: Anterior cruciate ligament
DPX: p-xylene-bis-pyridinium bromide
ECM: Extracellular matrix
INSL: Insulin-like peptides
MMPs: Matrix metalloproteinases
one-way ANOVA: One-way Analysis of Variance
PBS: Phosphate Buffered Saline
RXFP1: Relaxin family peptide receptor
RXFP2: Relaxin family peptide receptor 2
TIMPs: Tissue inhibitors of metalloproteases
