# Supplementary material for: Relaxin inhibits patellar tendon healing in rats: a histological and biochemical evaluation
Source: BMC Musculoskelet Disord. 2019 Jul 27;20:349. doi: 10.1186/s12891-019-2729-3 (PMC6661089; doi:10.1186/s12891-019-2729-3)
Supplement: Supplementary file 1 — Table S1. Histological scoring system. (DOCX 17 kb) [file 12891_2019_2729_MOESM1_ESM.docx]

**Supplementary Table S1**

Histological scoring system[1]

| Extracellular matrix (ECM) organization | Points |
| --- | --- |
| Wavy, compact and parallel arranged collagen fibers | 2 |
| In part compact, in part loose collagen fibers | 1 |
| Loosely composed collagen fibers with granulation tissue | 0 |
| Cell/matrix ratio |  |
| Physiological | 2 |
| Locally increased cell density | 1 |
| Increased cell density | 0 |
| Cell distribution |  |
| Homogeneous and physiological | 1 |
| Heterogeneous and cell clustering | 0 |
| Organization of repair tissue |  |
| Homogeneous | 2 |
| Locally heterogeneous | 1 |
| Whole tissue composition changed | 0 |
| Degenerative changes/tissue metaplasia |  |
| Non existing | 3 |
| Moderate formation of edema | 2 |
| Intense edema and gap formation | 1 |
| Assembly of cartilage or bone 0 | 0 |
| Vascularization |  |
| Hypo-vascularized | 1 |
| Hyper-vascularized | 0 |
| Inflammation |  |
| No inflammatory cells | 1 |
| Inflammatory cell types (neutrophils, macrophages and giant cells) | 0 |
| Max total | 12 |

**References**

1. Stoll C, John T, Conrad C, Lohan A, Hondke S, Ertel W, Kaps C, Endres M, Sittinger M, Ringe J: **Healing parameters in a rabbit partial tendon defect following tenocyte/biomaterial implantation**. *Biomaterials* 2011, **32**(21):4806-4815.
